# Efficacy and Safety of Emergent Transcatheter Aortic Valve Implantation in Patients with Acute Decompensated Aortic Stenosis: Systematic Review and Meta-Analysis

**DOI:** 10.1155/2021/7230063

**Published:** 2021-12-24

**Authors:** Ruochen Shao, Junli Li, Tianyi Qu, Xiaoying Fu, Yanbiao Liao, Mao Chen

**Affiliations:** ^1^Laboratory of Heart Valve Disease, West China Hospital, Sichuan University, Chengdu, China; ^4^Department of Cardiology, West China Hospital, Sichuan University, Chengdu, China; ^2^West China School of Medicine, Sichuan University, Chengdu, China; ^3^Department of Clinical Research Management, West China Hospital, Sichuan University, Chengdu, China

## Abstract

**Introduction:**

The aim of this systematic review and meta-analysis was to investigate the efficacy and safety of emergent transcatheter aortic valve implantation (TAVI) in patients with decompensated aortic stenosis (AS) by comparing the clinical outcomes with the patients who had received the elective TAVI.

**Methods:**

By searching PubMed, EMBASE, and Cochrane databases, we obtained the studies comparing the clinical outcomes of emergent TAVI and elective TAVI. Finally, 14 studies were included.

**Results:**

A total of 14 eligible articles with 73,484 patients were included in this meta-analysis. Emergent TAVI was associated with a higher mortality during hospitalization (HR 2.09, 95% CI [1.39 to 3.14]), 30 days (HR 2.29, 95% CI [1.69 to 3.10]), and 1 year (HR 1.96, 95% CI [1.55 to 2.49]). Consistently, the incidence of acute kidney injury (AKI) (RR 2.48, 95% CI [1.85 to 3.32]), dialysis (RR 2.37, 95% CI [1.95 to 2.88]), bleeding (RR 1.62, 95% CI [1.27 to 2.08]), major bleeding (RR 1.05, 95% CI [1.00 to 1.10]), and 30-day rehospitalization (RR 1.30, 95% CI [1.07, 1.58]) were more common in patients receiving emergent TAVI. No statistical differences were found in the occurrence rate of vascular complications (RR 1.11, 95% CI [0.90, 1.36]), major vascular complications (RR 1.14, 95% CI [0.52, 2.52]), permanent pacemaker (PPM) placement (RR 1.05, 95% CI [0.99, 1.11]), cerebrovascular events (RR 1.11, 95% CI [0.98, 1.25]), moderate to severe paravalvular leakage (PVL) (RR 1.23, 95% [CI 0.94 to 1.61]), and device success (RR 0.99, 95% CI [0.97, 1.01]).

**Conclusion:**

Emergent TAVI is associated with some postoperative complications and increased mortality compared with elective TAVI. Emergent TAVI should be implemented cautiously and individually.

## 1. Introduction

Aortic stenosis (AS) is one of the commonest valvular heart diseases, and its prevalence increased markedly with population aging [1, 2]. A conservative, multicenter registry reported that the 1-year mortality in the patient with severe AS and heart failure that treated conservatively could go as high as 40%, which was about 3 times that of patients without heart failure [3]. However, there were no guidelines to explicitly recommend the treatment of acute decompensated AS. TAVI provided a less invasive option for AS, which had comparable and sometimes superior results to surgical aortic valve replacement (SAVR), while there were no recommendations for TAVI to treat acute decompensated AS. Recently, several studies reported the application of emergent TAVI in acute decompensated AS. Therefore, we conducted a systematic review and meta-analysis to panoramically and quantitatively investigate the efficacy and safety of emergent TAVI by comparing the outcomes with elective TAVI in order to comprehensively illustrate the clinical outcomes of emergent TAVI.

## 2. Methods

The present systematic review and meta-analysis was performed in conformity to the Preferred Reporting Items for Systematic Reviews and Meta-Analyses (PRISMA) statement.

### 2.1. Registration

Our systematic review was registered online in INPLASY (registration number: 202140050, https://inplasy.com/inplasy-2021-4-0050/).

### 2.2. Eligibility Criteria

The inclusion criteria in this meta-analysis were ① reporting outcome indicators for both emergent TAVI and elective TAVI; ② randomized clinical trials and prospective/retrospective cohort studies; and ③ presenting the specific number or incidence of outcome indicators or displayed the survival curve. The exclusion criteria included ① non-English literature; ② repetitive published literature; ③ research that cannot extract or transform key data; and ④ certain publication type (e.g., case reports, reviews, meta-analysis, editorials, guidelines, and letters).

Our primary outcomes were mortality within hospitalization, 30 days, and 1 year after TAVI. The secondary outcomes included device success; rehospitalization within 30 days after TAVI; and procedural-related complications including acute kidney injury (AKI), the need for dialysis, bleeding, moderate to severe paravalvular leakage (PVL), vascular complications, permanent pacemaker (PPM) implantation, and cerebrovascular events including TIA and stroke, as defined in the original studies. When information about the outcome of interest is unavailable, this study was not analyzed for this endpoint.

### 2.3. Data Sources and Study Selection

A literature search of Embase, PubMed, and Cochrane Library was performed using following search strategies: ((emergent OR emergency OR urgent OR emergently OR urgently) AND (elective OR electively OR nonurgent) AND (Transcatheter Aortic Valve Implantation OR Transcatheter Aortic Valve Replacement Implantation OR TAVI OR TAVR)) OR eTAVI OR eTAVR. We also reviewed corresponding references of retrieved studies to identify relevant clinical trials that were neglected. The time interval of the search was from Jan. 2009 to Apr. 2021. The PRISMA diagram displays the process for the filtration and selecting of references.

### 2.4. Data Extraction and Quality Evaluation

Two researchers (SRC and LJL) independently screened literatures and extracted data. If under the circumstance of disagreement, it shall be settled through discussion or negotiation with a third party. After excluding the obviously unrelated articles, further read abstract and the full text to determine if they meet the inclusion criteria. If there were lack of information, researchers tried to contact the author as far as possible to acquire the relevant data. Data extraction included but was not limited to ① basic information of included studies: research topic, first author, published journal, etc.; ② baseline characteristic and intervention measure of research objects; ③ key element of bias risk assessment; and ④ outcome indicators concerned. If mortality or the number of deaths were not presented directly, the digitizing software Engauge Digitizer 4.1 was used to gain information from survival cure. The Newcastle–Ottawa Scale (NOS) was used to estimate the quality of included observational studies.

### 2.5. Statistical Analysis

Stata 15.1 software was used for statistical analysis. The risk ratio (RR) or hazard ratio (HR) was used as the statistic of effect analysis. The random effect model was used for meta-analysis. Heterogeneity was evaluated by calculating *I*^2^ statistic as well as its *P* value. Heterogeneity was appraised by the Galbraith radial plot, cumulative analysis, and sensitivity analysis. Meta-regression was also used to analyze the contribution of the remaining characteristics of each study to heterogeneity. A two-sided *P* value of 0.05 was considered statistically significant.

### 2.6. Publication Bias Analysis

The funnel plot was drawn and intuitively judged for the main outcome measures. If the distribution was a left-right symmetrical inverted funnel, it indicates that there was no remarkable publication bias in the study. Begg's rank correction test and Egger's linear regression were performed, and *P* < 0.05 indicates that there might be publication bias.

## 3. Results

### 3.1. Literature Search and Eligible Studies

**Figure 1:**
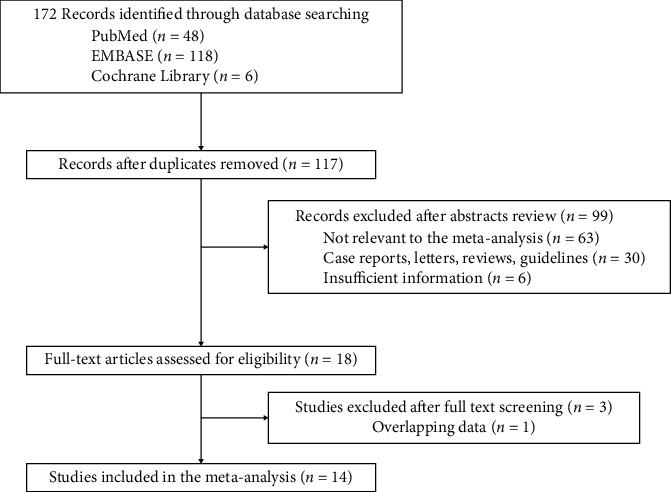
Flow diagram of the identification process for eligible studies.

The selection process details are illustrated in Figure 1. We initially retrieved a total of 172 citations from Cochrane Library, Embase, and PubMed, 55 of which were duplicated and removed and a total of 99 publications were excluded behind screening abstracts and titles. After reading the full text and excluding overlapping data, 14 retrospective cohort studies were finally included in our meta-analysis. The characteristics of the 14 studies and NOS assessments are summarized in Table 1. A range of 5–9 stars was gained, which indicates median to high methodological quality of the included studies. The baseline of the included overall studies is shown in Table 2.

### 3.2. Outcomes

#### 3.2.1. Mortality

**Figure 2:**
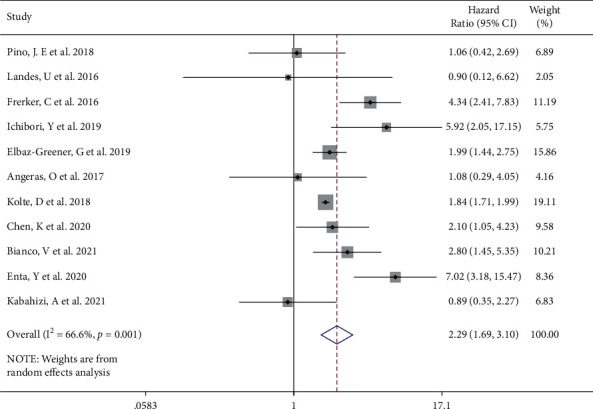
Forest plot showing the 30-day mortality of the emergent TAVI increased. CI, confidence interval.

**Figure 3:**
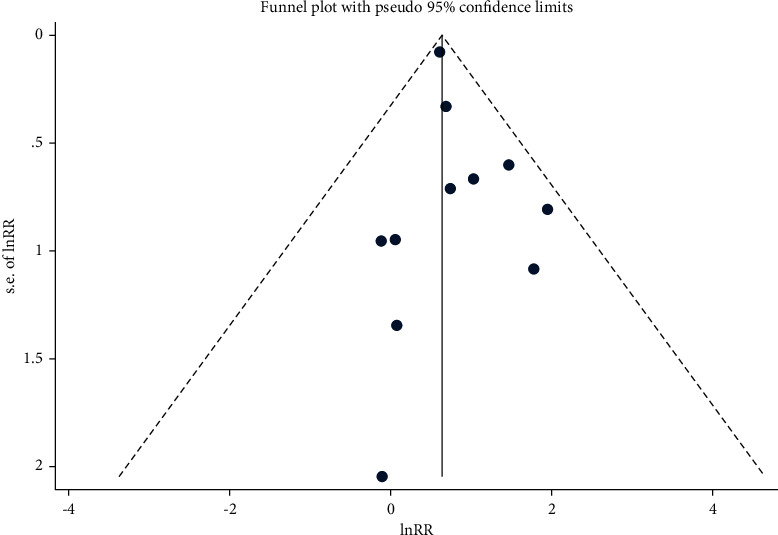
Funnel plot analysis of publication bias.

A total of 11 [4–14] studies reported all-cause 30-day mortality after TAVI in the emergent TAVI group and the elective TAVI group. The heterogeneity test (*I*^2^ = 66.6%, Q test *P*=0.001, Figure 2) indicated the significant heterogeneity between the selected literatures. The random effect model was used for combining the results. Emergent TAVI had significant higher 30-day mortality than elective TAVI (HR 2.29, 95% CI [1.69 to 3.10], Figure 2). The Galbraith radial plot (Supplemental Figure S1(a)) and cumulative meta-analysis (Supplemental Figure S1(b)) did not find a clear source of heterogeneity. Through sensitivity analysis of 11 literatures, we found that the research of Kolte et al. [10] might have an impact on heterogeneity (Supplemental Figure S1(c)). After deleting this study, the other 10 literatures showed the same trend as before, but there was still strong heterogeneity (Supplemental Figure S1(d)). Univariate meta-expression showed the sample size, gender, age, and basic health status of patients could not explain the source of heterogeneity (Supplemental Table 1). Besides, no evidence of publication bias was found according to funnel plots (Begg's test *P*=0.876 and Egger's test *P*=0.339, Figure 3).

**Figure 4:**
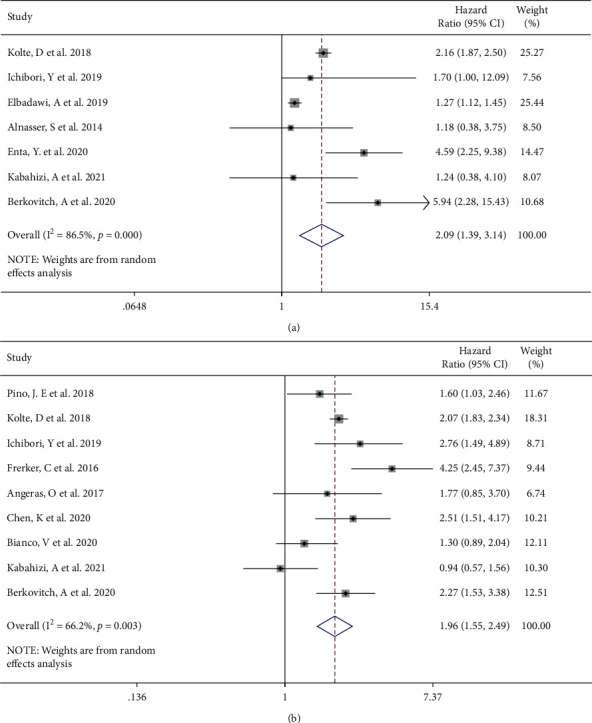
Forest plot showing a higher incidence of in-hospital (a) and 1-year (b) mortality of the emergent TAVI group. CI, confidence interval.

With regard to mortality during hospitalization and one year after TAVI, there were 7 [8, 10, 13–17] and 9 [4, 8, 10, 11, 13, 17] studies describing the pertinent data, respectively. A significant difference was noted between emergent and elective TAVI on the mortality at hospitalization (emergent vs selective, HR 2.09, 95% CI [1.39 to 3.14], *I*^2^ = 86.5%, Q test *P* < 0.001, Figure 4(a)) and 1 year (HR 1.96, 95% CI [1.55 to 2.49], *I*^2^ = 66.2%, Q test *P*=0.003, Figure 4(b)) in the random effect model. Cumulative meta-analysis and sensitivity analysis suggested the research of Elbadawi et al. [15] contributed to heterogeneity in the mortality at hospitalization (Supplemental Figures S2(a) and S2(b)). After excluding the study, emergent TAVI was still considered to have higher in-hospital mortality (emergent vs selective, HR 2.48, 95% CI [1.58 to 3.91], *I*^2^ = 52.2%, Q test *P*=0.063, Supplemental Figure S2(c)). Meta-regression did not find factors that might influence the results, in the case of sufficient original data support (Supplemental Table.  2). No exact source of heterogeneity was found in the Galbraith radial plot, cumulative meta-analysis, sensitivity analysis, and meta-regression of mortality at 1 year (Supplemental Figures S2(d)–S2(f), Supplemental Table 3). Begg's test and Egger's test for hospitalization mortality (Begg's test *P*=0.707, Egger's test *P*=0.483) and 1 year mortality (Begg's test *P*=0.602, Egger's test *P*=0.827) indicating no publication bias exist.

#### 3.2.2. Procedural Complications

**Figure 5:**
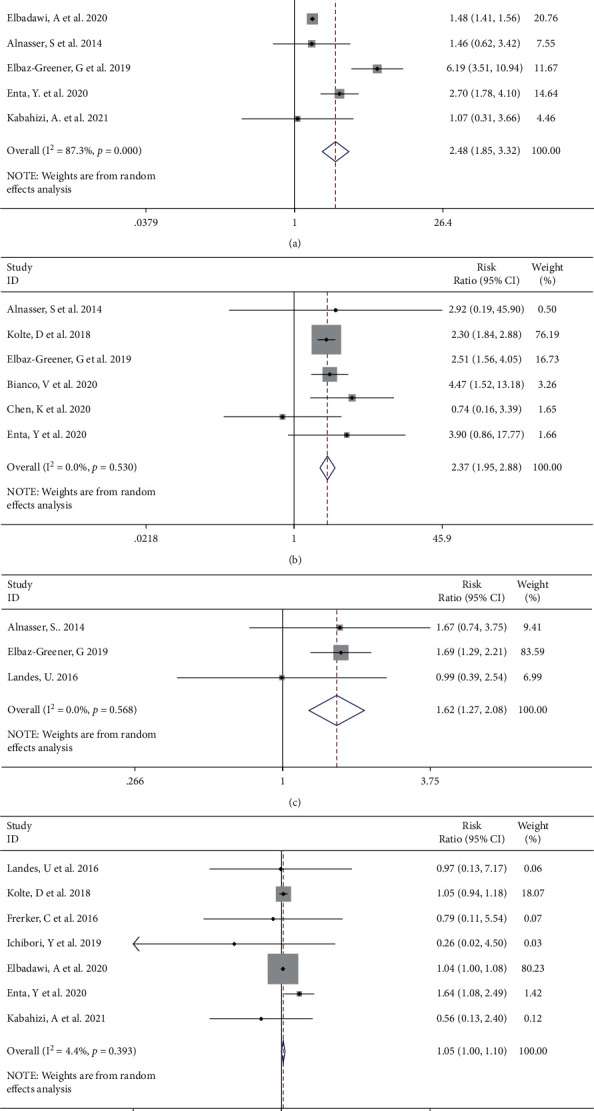
Forest plot showing the incidence of AKI (a), dialysis (b), bleeding (c), and major bleeding (d) increased in the emergent TAVI group. CI, confidence interval.

A variety of postoperative complications have been described in multiple studies. A total of 9 [4, 8, 10, 12–16] and 7 [6, 7, 9, 10, 14–16] studies investigated AKI and the need for dialysis after emergent TAVI. A random effect meta-analysis confirmed a statistically significant difference in opposition to the emergent TAVI from the aspects of AKI (RR 2.48, 95% CI [1.85 to 3.32], *I*^2^ = 87.3%, Q test *P* < 0.001, Figure 5(a)). After sensitivity analysis, we found that the study of Elbadawi et al. [15] had an impact on heterogeneity (Supplemental Figure S3(a)). After removing this study, the trend of effect quantity combined by meta-analysis did not change (Supplemental Figure S3(b)). In the aspect of dialysis, through sensitivity analysis, we found that the study of Elbadawi et al. [15] was a source of heterogeneity (Supplemental Figure S3(c)), so the observation was deleted. There was no statistical heterogeneity in the remaining studies (*I*^2^ = 0%, *P*=0.530); meta-analysis showed that emergent TAVI was inferior in the occurrence of dialysis (emergent vs selective, HR 2.37, 95% CI [1.95 to 2.88], Figure 5(b)). The prevalence rates of bleeding [9, 12, 16] (RR 1.62, 95% CI [1.27 to 2.08], *I*^2^ = 0.0%, Q test *P*=0.568, Figure 5(c)) and major bleeding [4, 8, 10, 12–15] (RR 1.05, 95% CI [1.00 to 1.10], *I*^2^ = 4.4%, Q test *P*=0.393, Figure 5(d)) were aggravated to a certain extent in the emergent TAVI group. Egger's test displayed evidence of the publication bias for AKI (*P*=0.034). The trim-and-fill analysis simulated and added 3 missing studies (RR 1.663, 95% CI [1.222, 2.261]). Begg's test and Egger's test found no evidence of publication bias for dialysis (Begg's test *P*=0.548, Egger's test *P*=0.397) and major bleeding (Begg's test *P*=0.548, Egger's test *P*=0.986).

**Figure 6:**
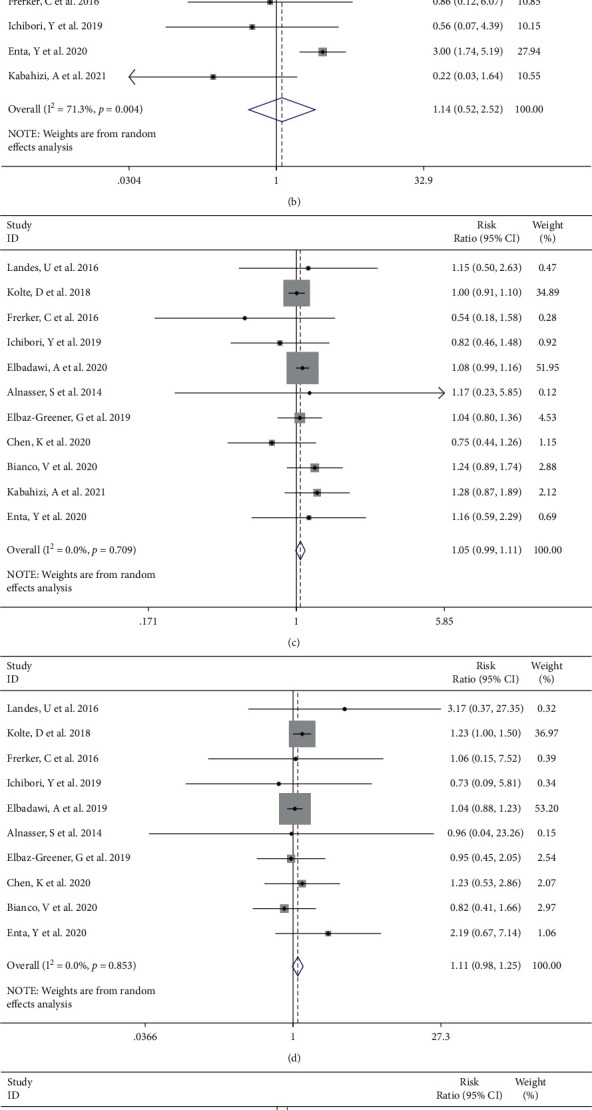
Forest plot showing there was no significant difference in vascular complications (a), major vascular complications (b), PPM placement (c), cerebrovascular events (d), and moderate to severe PVL (e) between the emergent TAVI group and elective TAVI group. CI, confidence interval.

Compared with those patients underwent elective TAVI, the emergent TAVI group showed no significant statistical difference in the incidence of vascular complications [4, 6, 8, 12, 15, 16] from random effects (RR 1.11, 95% CI [0.90 to 1.36], *I*^2^ = 15.1%, Q test *P*=0.317, Figure 6(a)). There was no significant difference from random effects in major vascular complications between two groups (RR 1.14, 95% CI [0.52 to 2.52], *I*^2^ = 71.3%, Q test *P*=0.004, Figure 6(b)). After removing one study with high heterogeneity through sensitivity analysis, the trend of the results did not change, and there was no decrease in heterogeneity (Supplemental Figures S3(d) and S3(e)). Consistently, there was no significant difference in the rates of PPM placement [4, 6, 10, 12–16] (RR 1.05, 95% CI [0.99 to 1.11], *I*^2^ = 0.0%, Q test *P*=0.709, Figure 6(c)) and cerebrovascular events [4, 6, 10, 12, 14–16] (RR 1.11, 95% CI [0.98 to 1.25], *I*^2^ = 0.0%, Q test *P*=0.853, Figure 6(d)). Moderate to severe PVL was described in 8 [4, 7, 8, 10, 12, 14, 16] studies. There was no statistically significant difference in opposition to the emergent TAVI group (RR 1.23, 95% CI [0.94 to 1.61], *I*^2^ = 18.1%, Q test *P*=0.287, Figure 6(e)). Egger's test and Begg's test suggested no evidence of publication bias for vascular complications (Egger's test *P*=0.089, Begg's test *P*=1.000), major vascular complications (Begg's test *P*=1.000, Egger's test *P*=0.558), PPM placement (Begg's test *P*=0.533, Egger's test *P*=0.666), cerebrovascular events (Begg's test *P*=0.592, Egger's test *P*=0.683), and moderate to severe PVL (Begg's test *P*=0.532, Egger's test *P*=0.907).

**Figure 7:**
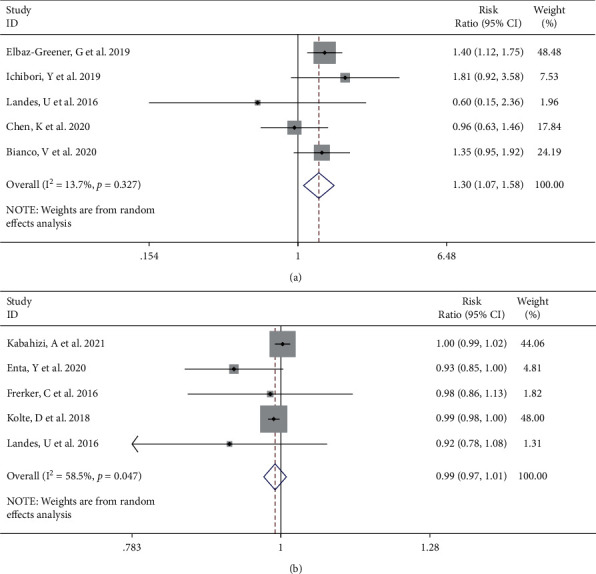
Forest plot showing rehospitalization rate (a) and device success (b) between the emergent TAVI group and elective TAVI group. CI, confidence interval.

The need for rehospitalization was described in 5 [6, 9, 12] studies. We found that emergent TAVI was associated with negative prognosis in terms of rehospitalization rate (RR 1.30, 95% CI [1.07 to 1.58], *I*^2^ = 13.7%, Q test *P*=0.327, Figure 7(a)). There were no statistically significant differences between the two groups in comparison of device success [4, 10, 12–14] (RR 0.99, 95% CI [0.97 to 1.01], *I*^2^ = 58.5%, Q test *P*=0.047, Figure 7(b)). Sensitivity analysis did not show a contribution to heterogeneity reduction (Supplemental Figures S3(f) and S3(g)).

## 4. Discussion

Main results of our systematic review and meta-analysis were as follows: (1) emergent TAVI was associated with higher incidence of 30-day, in-hospital, and 1-year mortality; and (2) in terms of postoperative adverse events, emergent TAVI had higher rates of AKI, dialysis, bleeding, and major bleeding. On the contrary, vascular complications, major vascular complications, PPM, cerebrovascular events, and moderate to severe PVL were comparable between emergent TAVI and elective TAVI.

Management of patients with acute decompensated AS remains challenging. Patients with acute decompensated aortic stenosis were usually not recommended for SAVR because of the high risk [10]. Standard medical therapy and balloon aortic valvuloplasty (BAV) alone was associated with various harmful outcomes after one year [18]. In this context, emergent TAVI and emergent BAV as the “bridge” for TAVI/SAVR have become the few optional strategies [19]. More and more studies have been proposed to investigate the safety and efficacy of emergent TAVI by comparing with elective TAVI conducted at the same time. The mortality after emergent TAVI reported in these original studies showed inconsistent trends. Our study pooled data derived from 14 articles finding the emergent TAVI in decompensated AS had higher risk for mortality before discharge and within 30 days and 1 year, in comparison with those stable AS undergoing elective TAVI. This reflects that emergent TAVI does not show absolute advantage in acute decompensation scenarios, although, in terms of some postoperative complications, emergent TAVI can achieve similar results as elective TAVI. As a more rapid and convenient rescue measure, BAV is also used as a buffer for decision-making, allowing patients to be reassessed in a more stable situation [20]. According to a recent single-center cohort study [21], there was no significant difference in 1-year mortality between patients receiving emergent TAVI and patients receiving TAVI or SAVR after emergent BAV. Further research should be conducted to individualize perioperative management to identify situations in which emergency TAVI benefits more and situations in which expanded use of BAV is warranted. Nowadays, the indications of TAVI are expanding [22] and TAVI is growing rapidly worldwide [23]. This may lead to longer wait times for TAVI patients to undergo surgery and greater risk for interval decompensation [6]. These findings emphasize the importance of optimizing the time from diagnosis to surgery, timely and correctly identifying the proneness of decompensated AS, and avoiding undesirable intervention conditions.

Our study showed that the incidence of bleeding and major bleeding after emergent TAVI increased, and major bleeding or life-threatening bleeding was associated with significant increase in the 30-day mortality [24]. The higher prevalence of bleeding might be explained by the high incidence of atrial fibrillation (AF) at baseline [25, 26] and higher incidence of AKI after emergent TAVI [27]. The increased incidence of baseline atrial fibrillation may be related to left ventricular pressure overload caused by AS [28]. Antithrombotic regimens, commonly administered to patients with atrial fibrillation, might be a crucial cause of the increased bleeding after TAVI [29]. Actually, a large proportion of patients required mechanical circulatory support and/or mechanical ventilation at the time of TAVI [30], which complicated the use of antithrombotic agents and increased the occurrence rate of bleeding.

The present study established that AKI and dialysis were more common after emergent TAVI. It was concerned that AKI and dialysis after TAVI impaired both early (in-hospital and 30-day) and late survival [27, 31, 32]. The risk factors of AKI were multifactorial. We observed preoperative risk factors [33] such as elevated baseline creatine and higher STS score in emergent TAVI patients. Patients with emergent TAVI might receive CT angiography and cardiac catheterization within a short time before surgery [10]. And contrast medium was considered an important factor of renal injury during TAVI [34]. Bleeding and postprocedural AR, which were more common in emergent TAVI patients, were also reported to be associated with AKI after TAVI [35]. There were no consensuses on AKI prevention in the TAVI patients. Furosemide-induced diuresis with matched isotonic intravenous hydration by the RenalGuard system is the effective device to reduce the happening of AKI in TAVI [36]. It helps to shorten the contact time between contrast medium and tubular cells without overloading the patient's volume [37]. There were also some case reports of patients who underwent TAVI with minimal contrast media during intraoperative and preoperative preparation. Arrigo et al. [38] carried out TAVI with the single contrast injection to ensure correct position of the pigtail catheter at the level of annulus. Then, the pigtail served as a marker for valve deployment. In a case report of Higuchi et al. [39], the valve was positioned using the calcified valve as a landmark, in which no contrast medium was used during TAVI. Although these individual cases cannot be generalized to all patients due to their limitations, they also provide ideas for preventing AKI in high-risk TAVI patients.

There are some limitations in our study. Firstly, some of our results had significant heterogeneity. Although we have used a variety of methods, we still cannot find the exact source of heterogeneity in some parts. Besides, our sensitivity analysis still demonstrated the robustness of a substantial part of our result. These parts of the results should be treated dialectically. Secondly, the studies in our meta-analysis were retrospective or prospective observational design. Confounding factors that were not included in the study cannot be excluded. Thirdly, despite our efforts to exclude overlapping data, there may still be overlapping data in our study due to inclusion of TVT studies. Finally, some abstracts were excluded due to the inability to obtain computable data, which had no definite impact on our study.

## 5. Conclusions

This meta-analysis investigated the differences in postoperative mortality and perioperative events between emergent and elective TAVI patients. Our study demonstrated that emergent TAVI was associated with increased 30-day, in-hospital, and 1-year mortality. Emergent TAVI increased the incidence of AKI and dialysis, bleeding, and major bleeding after TAVI; however, vascular complications, major vascular complications, PPM placement, cerebrovascular events, and moderate to severe PVL did not increase. The current results show that emergent TAVI is not able to achieve the same excellent results as elective TAVI. In the case of emergency, the decision-making of emergency TAVI should be individualized to avoid the occurrence of complications that may lead to the poor prognosis of emergent TAVI.

## Figures and Tables

**Table 1:** Characteristics and methodological quality assessments.

					Newcastle–Ottawa scale			
Study	Year	Location	Study design	Study period	Outcomes	Selection	Comparability	Outcome
Alnasser, S. et al	2014	Canada	Prospective cohort study	2009–2014	②④⑤⑦⑨⑩⑪⑭	★★★★	★☆	★★★
Landes, U. et al	2016	Israel	Prospective cohort study	2008.11–2015.4	①④⑥⑦⑧⑨⑩⑪⑫⑬⑭	★★★★	★☆	★★★
Frerker, C. et al	2016	Germany	Cohort study	2008.8–2013.9	①③④⑥⑧⑨⑩⑪⑫⑭	★★★★	★☆	★★☆
Angeras, O. et al	2017	Sweden	Cohort study	2008.5–2016.12	①③	★★☆★	☆☆	★★☆
Kolte, D. et al	2018	USA	Retrospective cohort study	2011.11–2016.6	①②③④⑤⑥⑧⑩⑪⑫⑭	★★★★	★★	★★☆
Pino, J.E. et al	2018	USA	Retrospective cohort study	—	①③	★★★★	☆☆	★★☆
Ichibori, Y. et al	2019	USA	Retrospective cohort study	2014.4–2017.3	①②③④⑥⑧⑨⑩⑪⑬⑭	★★★★	★☆	★★☆
Elbaz-Greener, G. et al	2019	Canada	Retrospective cohort study	2010.4–2016.3	①④⑤⑥⑦⑩⑪⑬	★★★★	★☆	★★★
Elbadawi, A. et al	2020	USA	Retrospective cohort study	2011–2014	②④⑤⑥⑨⑩⑪	★★★★	★★	★☆★
Chen, K. et al	2020	USA	Retrospective cohort study	2012.4–2017.7	①③⑤⑨⑩⑪⑬	★★★★	★☆	★★☆
Bianco, V. et al	2020	USA	Retrospective cohort study	2011–2018	①③⑤⑩⑪⑬⑭	★★★★	★★	★★★
Enta, Y. et al	2020	Japan	Retrospective cohort study	2013.10–2016.7	①②③④⑤⑥⑧⑩⑪⑫⑭	★★★★	★☆	★★★
Berkovitch, A. et al	2020	Israel	Prospectively cohort study	—	②③	★★☆★	★☆	★★☆
Kabahizi, A. et al	2021	UK	Retrospective cohort study	2007–2019	①②③④⑥⑧⑩⑫⑭	★★★★	★☆	★★★

Outcomes: ① 30-day mortality; ② in-hospital mortality; ③ one-year mortality; ④ AKI; ⑤ new dialysis; ⑥ major bleeding; ⑦ major and minor bleeding; ⑧ major vascular complication; ⑨ major and minor vascular complications; ⑩ PPM placement; ⑪ cerebrovascular events; ⑫ device success; ⑬ rehospitalization; ⑭ moderate to severe PVL.

**Table 2:** Baseline characteristics of patients in the emergent TAVI group and elective TAVI group.

Study	Sample size	Age, y	Female sex	STS score	Diabetes mellitus	Hypertension	Smoke	COPD	Chronic kidney injury	Dialysis-dependent	Previous stroke or TIA	Previous myocardial infarction	Previous CABG	Atrial fibrillation	Previous PCI	Peripheral vascular disease	NYHA functional class III/IV	Aortic valve area	Mean transvalvular aortic pressure gradient	LVEF
Landes, U. et al	Elective TAVI 342	82.1 ± 6.2	192	7.3 ± 4.5	111	314	—	73	103	—	61	29	68	106	—	54	325	0.63 ± 0.18	51 ± 18	—
	Emergent TAVI 27	80.1 ± 9.7	15	9.7 ± 6.1	13	25	—	5	15	—	8	4	6	12	—	6	27	0.59 ± 0.19	52 ± 8.8	—

Kolte, D. et al	Elective TAVI 36,090	83.3 ± 7.4	17474	6.45 ± 3.7	12690	32477	1672	—	—	1195	7520	8253	10212	14664	12531	10948	28641	—	—	57.7 ± 11.1
	Emergent TAVI 3952	83.3 ± 7.4	1902	12.5 ± 7.6	1557	3608	231	—	—	300	887	1323	1000	2002	1395	1302	3656	—	—	49.8 ± 17.1

Ichibori, Y. et al	Elective TAVI 369	81.6 ± 8.5	188	6.4 ± 3.6	160	—	17	97	—	12	55	85	100	135	148	74	268	0.74 ± 0.18	40.8 ± 15.5	53.6 ± 12.9
	Emergent TAVI 78	80.8 ± 9.6	38	7.4 ± 4.3	35	—	6	16	—	8	16	19	14	36	27	15	60	0.65 ± 0.17	42.5 ± 16.9	48.2 ± 15.5

Frerker, C. et al	Elective TAVI 744	80 ± 7	395	—	219	620	—	120	283	—	101	—	—	320	—	166	—	0.8 ± 0.3	38.4 ± 15.6	52.4 ± 13.2
	Emergent TAVI 27	78 ± 9	15	—	11	22	—	6	17	—	4	—	—	14	—	9	—	0.7 ± 0.2	35.1 ± 16.8	39.5 ± 15.4

Elbadawi, A. et al	Elective TAVI 10,121	80.6 ± 8.85	4768	—	3699	7978	2987	—	3792	—	—	1354	2237	—	2027	2968	—	—	—	—
	Emergent TAVI 10089	81.0 ± 9.05	5072	—	3470	7826	2413	—	4207	—	—	1230	1897	—	1713	2953	—	—	—	—

Alnasser, S. et al	Elective TAVI 152	83 ± 7	—	8.6 ± 4.7	42	132	—	—	—	5	—	37	48	25	—	19	139	0.59 ± 0.18	51.8 ± 15.6	55.0 ± 10.7
	Emergent TAVI 52	85.75 ± 5.19	—	7.1 ± 4.9	15	42	—	—	—	3	—	21	15	9	—	9	52	0.65 ± 0.16	49.0 ± 16.7	49.9 ± 13.8

Elbaz-Greener, G. et al	Elective TAVI 1741	81.9 ± 7.2	804	—	780	1644	—	618	168	51	—	—	424	442	617	94	—	—	—	—
	Emergent TAVI 429	81.3 ± 8.9	192	—	211	395	—	159	75	22	—	—	97	137	121	25	—	—	—	—

Chen, K. et al	Elective TAVI 463	85.3 ± 5.9	192	5.4 ± 2.8	158	414	14	174	—	—	46	136	109	218	—	120	376	0.64 ± 0.22	44.4 ± 11.2	55.6 ± 14.9
	Emergent TAVI 139	84.9 ± 6.7	59	7 ± 3.2	54	128	5	50	—	—	17	43	37	66	—	28	120	0.64 ± 0.22	46.4 ± 12.7	50 ± 15

Bianco, V. et al	Elective TAVI 946	82.6 ± 6.7	484	—	387	844	54	77	—	32	222	348	245	388	327	345	680	—	48.5 ± 13.4	—
	Emergent TAVI 247	81.3 ± 8.9	116	—	109	220	28	35	—	22	56	118	72	120	91	83	231	—	49.3 ± 18.3	—

Enta, Y. et al	Elective TAVI 1526	84.3 ± 5.0	1075	6.8 ± 3.4	401	1203	37	282	—	—	211	103	111	308	404	219	740	0.64 ± 0.17	50.5 ± 18.0	58.5 ± 11.9
	Emergent TAVI 87	84.9 ± 7.0	61	14.3 ± 9.6	29	65	7	15	—	—	20	13	9	31	27	27	77	0.56 ± 0.15	50.2 ± 20.0	47.9 ± 16.1

Kabahizi, A. et al	Elective TAVI 975	82.0 ± 2.1	455	4.56 ± 2.69	176	—	—	198	121	16	—	—	140	187	—	229	—	—	—	—
	Emergent TAVI 182	80.1 ± 7.0	77	5.79 ± 4.13	40	—	—	34	34	3	—	—	22	42	—	42	—	—	—	—

STS: Society of Thoracic Surgeons Cardiac Surgery Risk Models, COPD: chronic obstructive pulmonary disease, PCI: percutaneous coronary intervention, NYHA: New York Heart Association, LVEF: left ventricular ejection fractions, CABG: coronary artery bypass graft, TIA: transient ischemia attack. Continuous variables were expressed as means ± SD.

## Data Availability

The figures and tables data used to support the findings of this study are included within the article.

## Supplementary Materials

Supplementary Materials Supplemental Figure S1: Galbraith radial plot (a), cumulative meta-analysis (b), and sensitivity analysis (c) showing the contribution of results from the 11 studies to heterogeneity. Forest plot showing the 30-day mortality after removing an article with heterogeneity (d). Supplemental Figure S2: Cumulative meta-analysis (a) and sensitivity analysis (b) showing the contribution of results from the 7 studies to heterogeneity. Forest plot showing the in-hospital mortality after removing an article with heterogeneity (c). Galbraith radial plot (d), cumulative meta-analysis (e), and sensitivity analysis (f) showing the contribution of results from the 9 studies to heterogeneity. Supplemental Figure S3: Sensitivity analysis (a) showing the contribution of results from the 9 studies to heterogeneity. Forest plot showing the incidence of AKI after removing an article with heterogeneity (b). Sensitivity analysis (c) showing the contribution of results from the 7 studies to heterogeneity. Sensitivity analysis (d) showing the contribution of results from the 6 studies to heterogeneity. Forest plot showing the incidence of major vascular complications after removing an article with heterogeneity (e). Sensitivity analysis (f) showing the contribution of results from the 5 studies to heterogeneity. Forest plot showing the incidence of device success after removing an article with heterogeneity (g). Supplemental Table 1: Univariate meta-regression on 30-day mortality of emergent TAVI and selective TAVI. Supplemental Table 2: Univariate meta-regression on mortality during hospitalization of emergent TAVI and selective TAVI. Supplemental Table 3: Univariate meta-regression on 1-year mortality of emergent TAVI and selective TAVI. .
