# Supplementary material for: Efficacy and Safety of Emergent Transcatheter Aortic Valve Implantation in Patients with Acute Decompensated Aortic Stenosis: Systematic Review and Meta-Analysis
Source: J Interv Cardiol. 2021 Dec 24;2021:7230063. doi: 10.1155/2021/7230063 (PMC8719985; doi:10.1155/2021/7230063)
Supplement: Supplementary Materials — Supplemental Figure S1: Galbraith radial plot (a), cumulative meta-analysis (b), and sensitivity analysis (c) showing the contribution of results from the 11 studies to heterogeneity. Forest plot showing the 30-day mortality after removing an article with heterogeneity (d). Supplemental Figure S2: Cumulative meta-analysis (a) and sensitivity analysis (b) showing the contribution of results from the 7 studies to heterogeneity. Forest plot showing the in-hospital mortality after removing an article with heterogeneity (c). Galbraith radial plot (d), cumulative meta-analysis (e), and sensitivity analysis (f) showing the contribution of results from the 9 studies to heterogeneity. Supplemental Figure S3: Sensitivity analysis (a) showing the contribution of results from the 9 studies to heterogeneity. Forest plot showing the incidence of AKI after removing an article with heterogeneity (b). Sensitivity analysis (c) showing the contribution of results from the 7 studies to heterogeneity. Sensitivity analysis (d) showing the contribution of results from the 6 studies to heterogeneity. Forest plot showing the incidence of major vascular complications after removing an article with heterogeneity (e). Sensitivity analysis (f) showing the contribution of results from the 5 studies to heterogeneity. Forest plot showing the incidence of device success after removing an article with heterogeneity (g). Supplemental Table 1: Univariate meta-regression on 30-day mortality of emergent TAVI and selective TAVI. Supplemental Table 2: Univariate meta-regression on mortality during hospitalization of emergent TAVI and selective TAVI. Supplemental Table 3: Univariate meta-regression on 1-year mortality of emergent TAVI and selective TAVI. . [file 7230063.f1.docx]

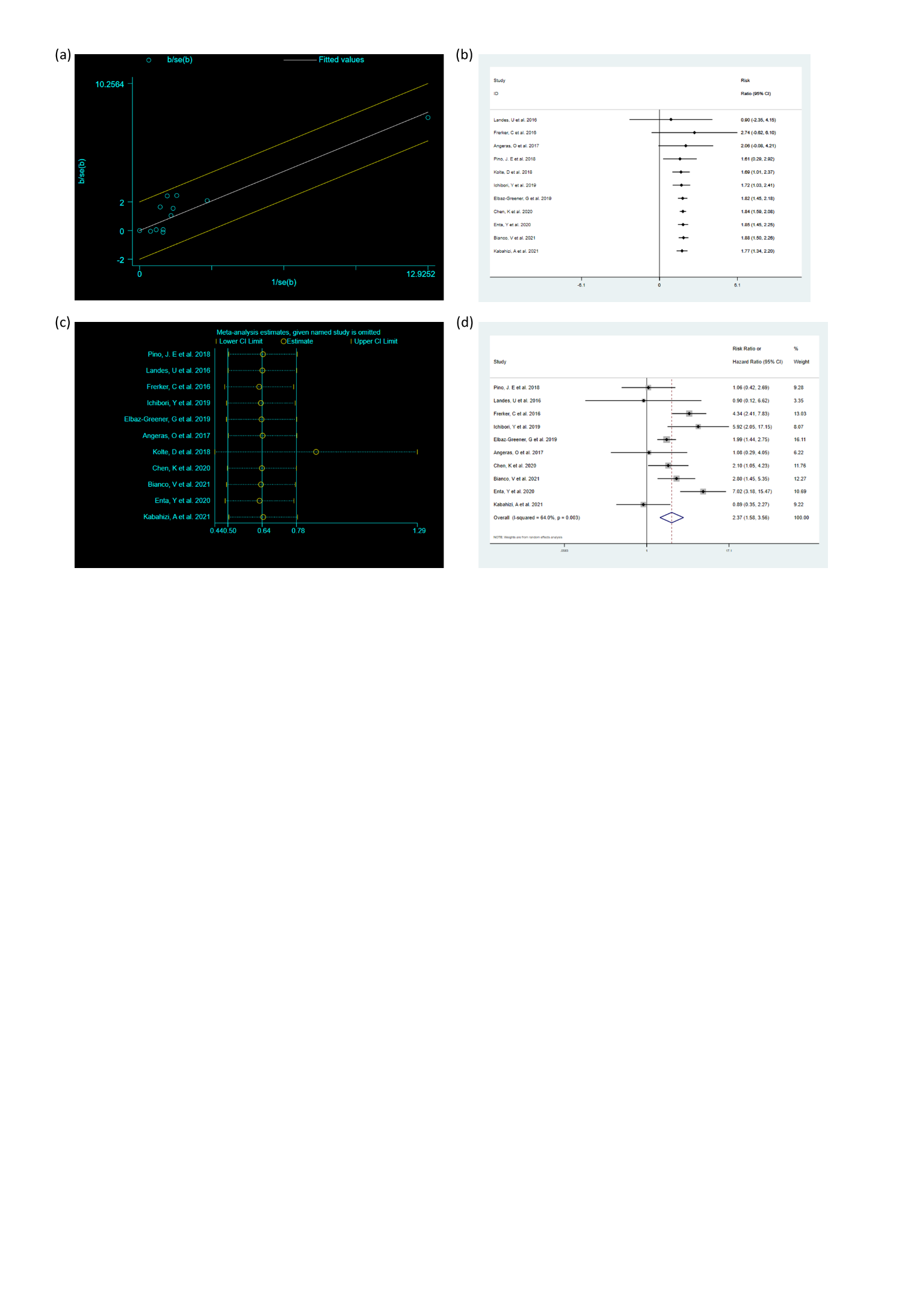


Supplemental Figure S1. Galbraith radial plot (a), cumulative meta-analysis (b) and sensitivity analysis (c) showing the contribution of results from the 11 studies to heterogeneity. Forest plot showing the 30-day mortality after removing an article with heterogeneity (d).


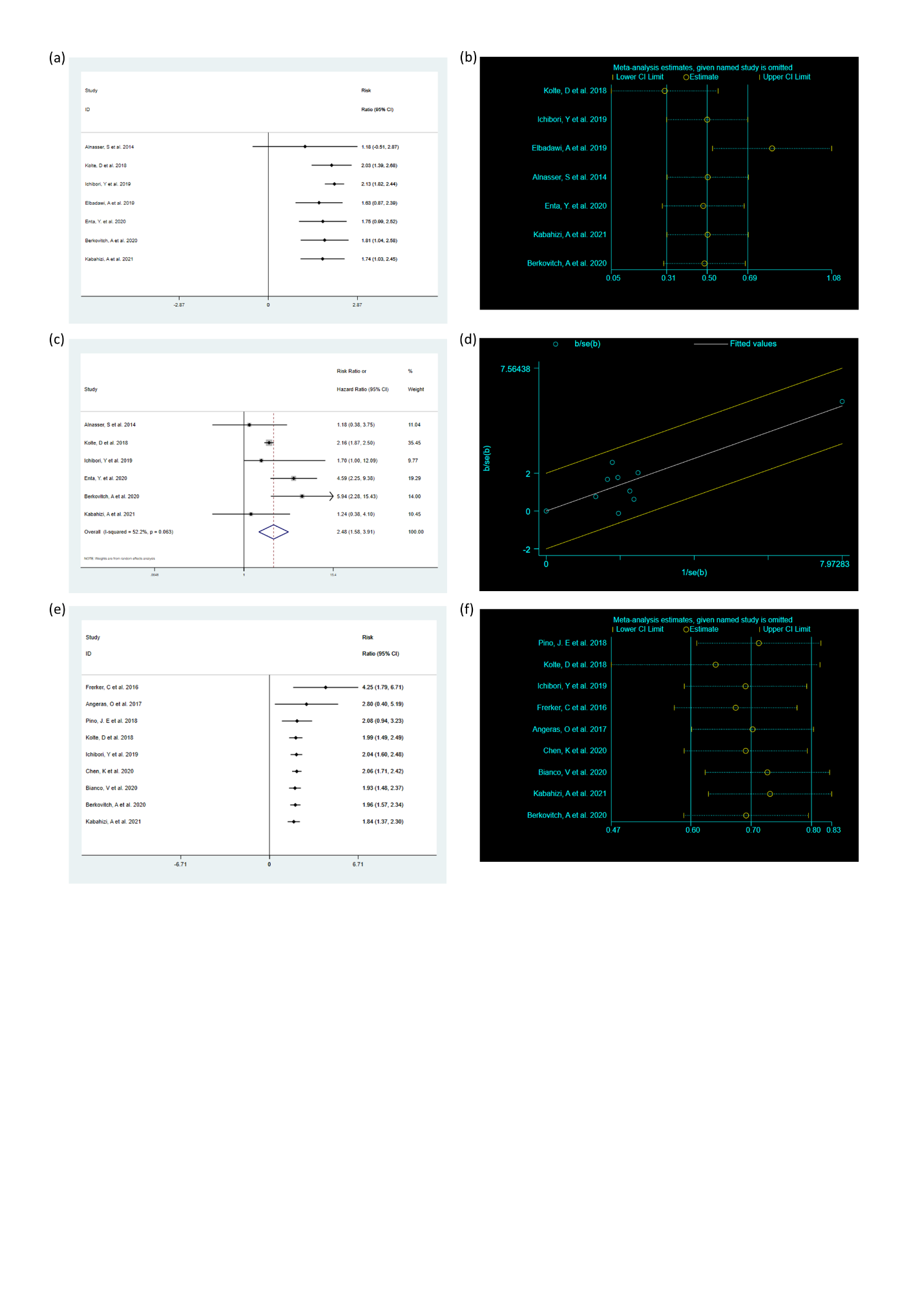


Supplemental Figure S2. Cumulative meta-analysis (a) and sensitivity analysis (b) showing the contribution of results from the 7 studies to heterogeneity. Forest plot showing the in-hospital mortality after removing an article with heterogeneity (c). Galbraith radial plot (d), cumulative meta-analysis (e) and sensitivity analysis (f) showing the contribution of results from the 9 studies to heterogeneity.


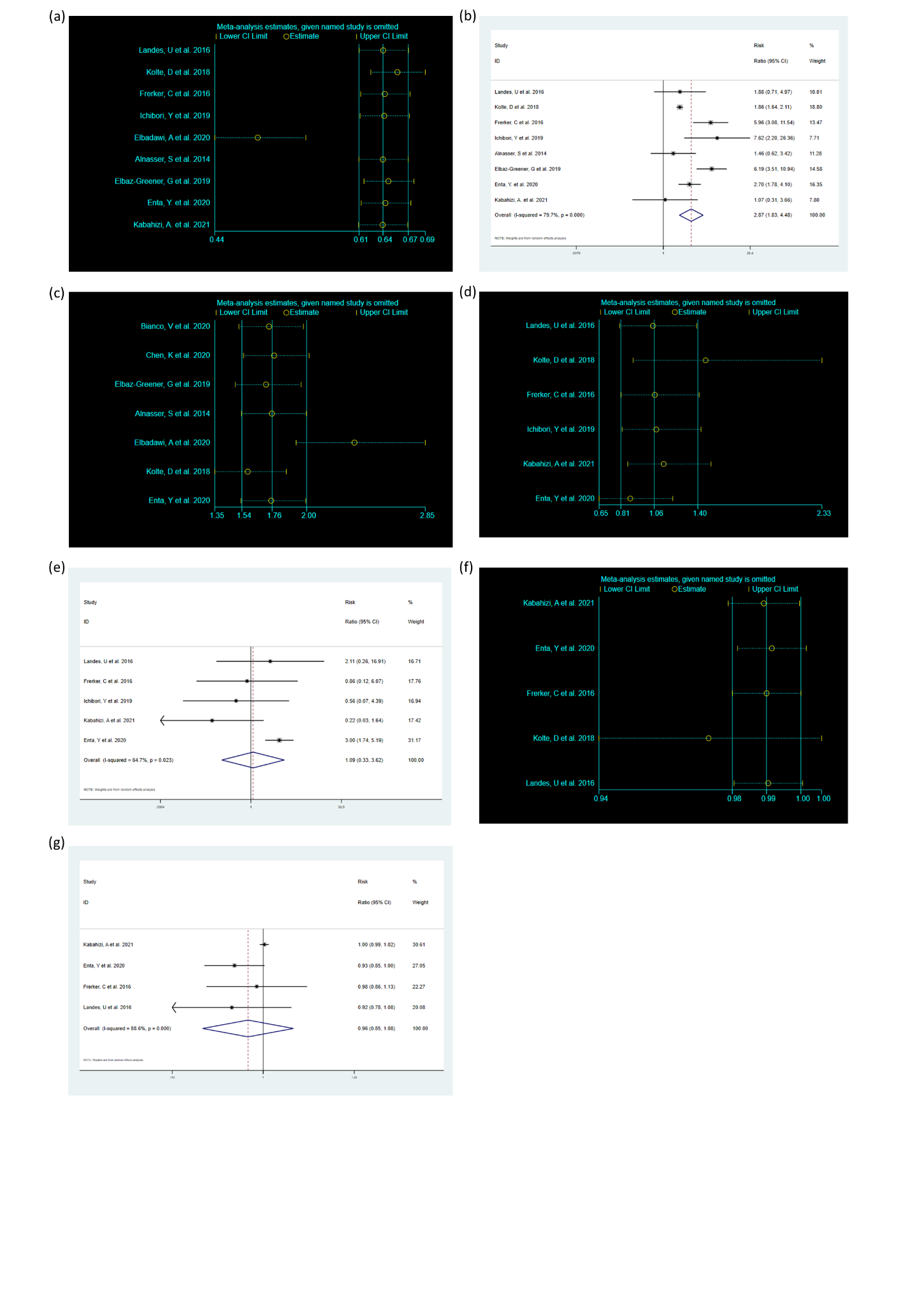


Supplemental Figure S3. sensitivity analysis (a) showing the contribution of results from the 9 studies to heterogeneity. Forest plot showing the incidence of AKI after removing an article with heterogeneity (b). Sensitivity analysis (c) showing the contribution of results from the 7 studies to heterogeneity. Sensitivity analysis (d) showing the contribution of results from the 6 studies to heterogeneity. Forest plot showing the incidence of major vascular complications after removing an article with heterogeneity (e). Sensitivity analysis (f) showing the contribution of results from the 5 studies to heterogeneity. Forest plot showing the incidence of device success after removing an article with heterogeneity (g).

| Characteristics | P Value |
| --- | --- |
| Sample Size | 0.379 |
| Age | 0.799 |
| Proportion of female | 0.641 |
| STS score | 0.886 |
| Diabetes mellitus | 0.468 |
| Hypertension | 0.881 |
| COPD | 0.587 |
| Previous Stroke or TIA | 0.963 |
| Previous Myocardial Infarction | 0.764 |
| Atrial Fibrillation | 0.766 |
| Peripheral Vascular Disease | 0.488 |
| NYHA Class III/IV | 0.914 |
| LVEF | 0.907 |

Supplemental Table 1. Univariate meta-regression on 30-day mortality of emergent TAVI and selective TAVI.

| Characteristics | P Value |
| --- | --- |
| Sample Size | 0.507 |
| Age | 0,412 |
| Proportion of female | 0.356 |
| STS score | 0.809 |
| Diabetes mellitus | 0.906 |
| Atrial Fibrillation | 0.827 |
| Peripheral Vascular Disease | 0.694 |

Supplemental Table 2. Univariate meta-regression on mortality during hospitalization of emergent TAVI and selective TAVI.

| Characteristics | P Value |
| --- | --- |
| Sample Size | 0.718 |
| Age | 0.935 |
| Proportion of female | 0.612 |
| Diabetes mellitus | 0.574 |
| COPD | 0.588 |
| Previous Stroke or TIA | 0.276 |
| Atrial Fibrillation | 0.203 |
| Peripheral Vascular Disease | 0.496 |

Supplemental Table 3. Univariate meta-regression on 1 year mortality of emergent TAVI and selective TAVI.
